# Single-nucleus transcriptomic analysis reveals the relationship between gene expression in oligodendrocyte lineage and major depressive disorder

**DOI:** 10.1186/s12967-023-04727-x

**Published:** 2024-01-27

**Authors:** Yinping Xie, Lijuan Chen, Leimin Wang, Tongou Liu, Yage Zheng, Lujia Si, Hailong Ge, Hong Xu, Ling Xiao, Gaohua Wang

**Affiliations:** 1 Institute of Neuropsychiatry, Renmin Hospital of Wuhan University, Wuhan, China; 2 Agricultural Genomics Institute at Shenzhen, Chinese Academy of Agricultural Sciences, Shenzhen, China; 3 School of Automation, China University of Geosciences, Wuhan, China; 4 The First Clinical College of Hubei University of Chinese Medicine, Wuhan, China; 5 Judicial Appraisal Institute, Renmin Hospital of Hubei Province, Wuhan, China; 6 Department of Psychiatry, Renmin Hospital of Wuhan University, Wuhan, China

**Keywords:** Major depressive disorder, Oligodendrocyte lineage development, Single-cell transcriptome, Guided regularized random forest algorithm, *MALAT1*

## Abstract

**Background:**

Major depressive disorder (MDD) is a common mental illness that affects millions of people worldwide and imposes a heavy burden on individuals, families and society. Previous studies on MDD predominantly focused on neurons and employed bulk homogenates of brain tissues. This paper aims to decipher the relationship between oligodendrocyte lineage (OL) development and MDD at the single-cell resolution level.

**Methods:**

Here, we present the use of a guided regularized random forest (GRRF) algorithm to explore single-nucleus RNA sequencing profiles (GSE144136) of the OL at four developmental stages, which contains dorsolateral prefrontal cortex of 17 healthy controls (HC) and 17 MDD cases, generated by Nagy C et al. We prioritized and ordered differentially expressed genes (DEGs) based on Nagy et al., which could predominantly discriminate cells in the four developmental stages and two adjacent developmental stages of the OL. We further screened top-ranked genes that distinguished between HC and MDD in four developmental stages. Moreover, we estimated the performance of the GRRF model via the area under the curve value. Additionally, we validated the pivotal candidate gene *Malat1* in animal models.

**Results:**

We found that, among the four developmental stages, the onset development of OL (OPC2) possesses the best predictive power for distinguishing HC and MDD, and long noncoding RNA *MALAT1* has top-ranked importance value in candidate genes of four developmental stages. In addition, results of fluorescence in situ hybridization assay showed that *Malat1* plays a critical role in the occurrence of depression.

**Conclusions:**

Our work elucidates the mechanism of MDD from the perspective of OL development at the single-cell resolution level and provides novel insight into the occurrence of depression.

**Supplementary Information:**

The online version contains supplementary material available at 10.1186/s12967-023-04727-x.

## Introduction

Major depressive disorder (MDD) is a heterogeneous disease with multiple causes that affects an estimated 350 million people of all ages worldwide [1]. Research related to neuropsychiatric disorders such as MDD has dominantly focused on neurons [2]. Glia make up roughly half of the total cells in the central nervous system (CNS). However, glia have been considered static bystanders in the formation and function of the CNS [3]. Glia and neurons were first described during the same period, but due to the limitations of research methods, the study of glial cells remained at the morphological level for a long time [4]. A deep insight of glial cells could contribute to understanding of mental illness.

During the past two decades, oligodendrocyte progenitor cells (OPCs) have become known as the fourth member of the glia family, in addition to astrocytes, microglia, and oligodendrocytes [5]. It is universally acknowledged that the human brain comprises 3–10% OPCs, 25% oligodendrocytes, 20% astrocytes, and 5–15% microglia [6–8]. OPCs are widely distributed in the adult brain and are the most proliferative cell type in the adult CNS [9]. They differentiate and mature into oligodendrocytes during development as well as throughout adulthood. Differentiation of OPCs into oligodendrocytes follows a complex, multistep, tightly regulated process [9]. OPCs and oligodendrocytes are uniformly referred to as the oligodendrocyte lineage (OL). There is growing evidence that the OL is not just a “passive supporter” of neurons, and the cells comprising the OL are now recognized as metabolic exchangers of neurons, a cellular interface of blood vessels and responders to gut-derived metabolites or changes in the social environment [10]. Both preclinical [11–14] and clinical [15, 16] studies have shown that the OL plays an important role in the pathogenesis of depression. Therefore, studying the development of the OL is of great importance.

Studies have suggested that patients with depression suffer damage to multiple brain regions, including the hippocampus, prefrontal cortex, amygdala and hypothalamus [17, 18]. In addition to the complexity of the brain regions involved, each brain region comprises multiple types of cells with different neural circuits [19]. Each cell type acts differently and has intricate interactions with others. Given the complicated structure and multitudinous cell types of the brain, there is a high probability of the low abundance of some cell types and transcripts [20, 21]. More in-depth and accurate research approaches will pave the way for studying an elusive mental illness such as MDD [22, 23].

The rapid development of sequencing technology has occurred during the twenty-first century. The invention of single-cell RNA sequencing (scRNA-seq) technology has enabled a breakthrough in research involving the molecular mechanism profiling of many inherently complex diseases [24]. In conventional bulk RNA-sequencing (RNA-seq) analysis, the final signal is actually the average of signals from numerous cells from different regions and/or different cell types. As a result, much of the cell type-specific information is usually overlooked. For example, when a transcript has low abundance, it is very difficult to judge whether it is highly expressed in a rare cell type or expressed at low levels in most of the dominant cell types. Unlike bulk RNA-seq, scRNA-seq, a revolutionary tool, enables the analysis of transcriptomes at single-cell resolution. Since the first publication of an scRNA-seq study, this technique has been widely used in biomedical research in many contexts, including the study of tumour heterogeneity [25, 26], the identification of new cell types [27, 28], the study of tissue development and cell differentiation [29, 30], studies of gene regulatory network (GRN) [31], and investigations of differences in allelic gene expression [32].

Although single-cell sequencing technology conveniently addresses research heterogeneity, subsequent data analysis is also a problem that cannot be ignored. Due to the temporal and spatial specificity of gene expression in cell types, many genes are not expressed in a certain type of cell at a certain time, which leads to node sparsity issues when creating tree-based predictive models. When instances are recursively split in the tree, the number of instances decreases, and measures calculated from nodes with a spot of instances cannot effectively distinguish between features with different predictive information. The extraction of significant features contributes to implement the efficient model, improve the accuracy and reduce the training time. An ensemble machine learning algorithm, random forest (RF), comprises decision trees, which is quick and robust to the noise of target data [33].

Guided regularized random forest (GRRF) is an enhanced method for RF analysis, which is computationally efficient and compact feature subsets [34]. GRRF obtains a subset of relevant and non-redundant features by the regularization of the information gain in the random forest nodes [35]. The importance scores in GRRF are from a preliminary RF, and each feature in the RF is assigned a penalty coefficient. GRRF model couple with prior or statistical information to well select feature. Previous study demonstrated that integration of statistical feature extraction and GRRF feature selection can enhance the detection accuracy compared to conventional detection methods [33]. GRRF is constantly applied to analyse highly heterogeneous data, GRN, integrated multiple data and single-cell sequencing data [36–39].

In this study, we attempt to illustrate the relationship between OL development and depression at the single-cell level using GRRF methods. We addressed the following three problems, which are listed in order of increasing complexity. (1) Which genes distinguish the four stages of development? (2) Which genes promote the transformation of two adjacent developmental stages? (3) Which of the four developmental stages has better predictive power of MDD? Our work links the development of the OL with the occurrence of depression, providing a new perspective for the study of the pathogenesis of depression.

## Materials and methods

### Single-nucleus RNA-Seq data collection

**Fig. 1:**
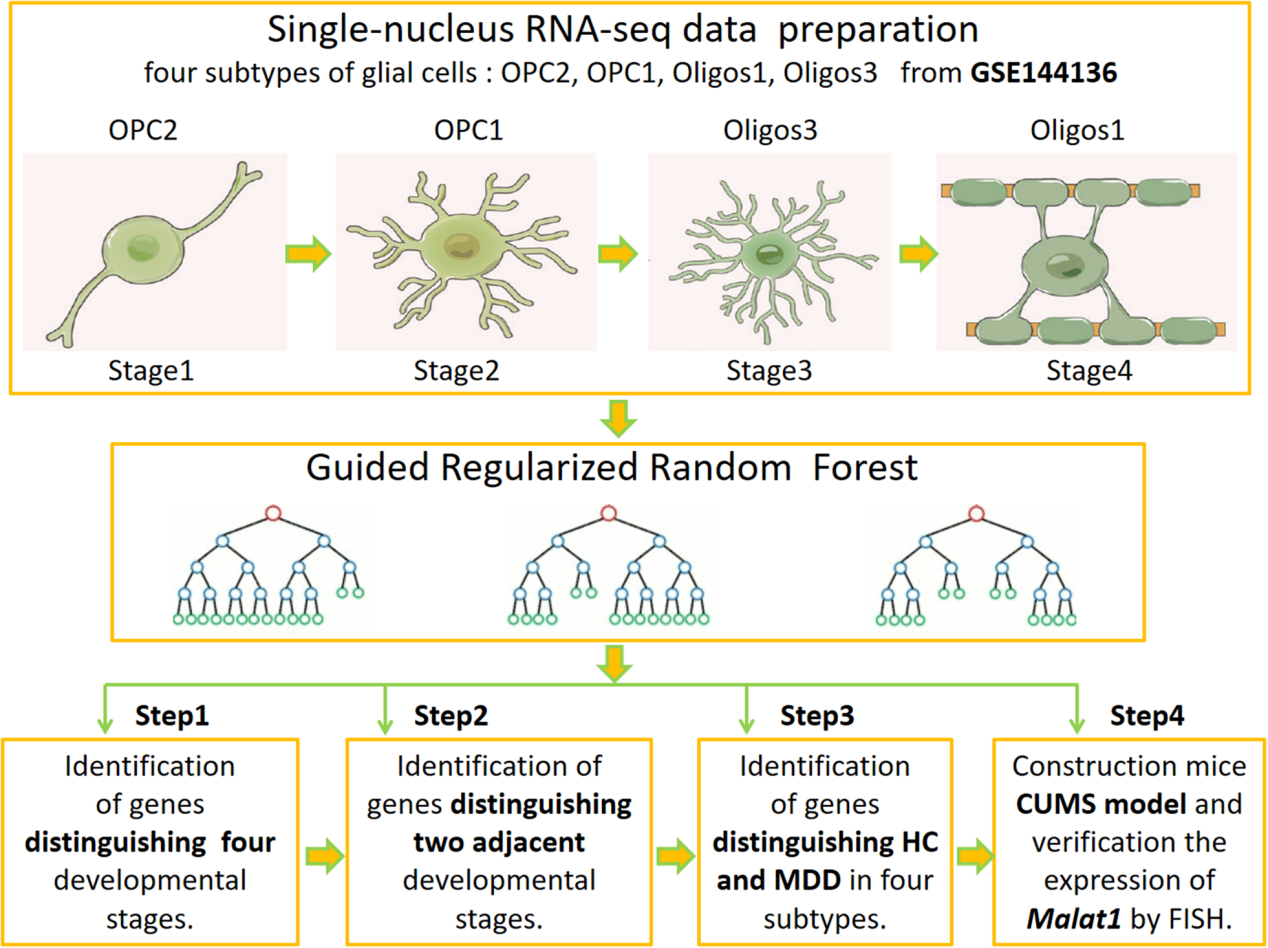
Data analysis flow chart. OPC, oligodendrocyte progenitor cell; Oligos, oligodendrocytes; HC, healthy control; MDD, major depressive disorder

1. The GSE144136 dataset was downloaded from the Gene Expression Omnibus database. GSE144136 contains postmortem dorsolateral prefrontal cortex (BA9) tissue from 17 healthy controls (HC) and 17 MDD cases who died by suicide [13]. All subjects were male. The sample collection process lasted nearly 15 years, and single-nucleus RNA sequencing (snRNA-seq) was performed on the frozen samples.
2. A total of 26 distinct cell types were identified in GSE144136, among which are five cell types belonging to the OL, namely, OPC1, OPC2, oligodendrocytes1, oligodendrocytes2, and oligodendrocytes3 (Hereafter, oligodendrocytes are designated as “Oligos”). Four cell types were selected: OPC1, OPC2, Oligos1, and Oligos3. After quality control filtering, there were merely few Oligos2 cells, which was inappropriate for constructing prediction model. Therefore, this cell type was excluded from this study. The numbers of the four cell clusters in the HC and MDD groups are displayed in Table 1. We downloaded the differentially expressed genes (DEGs) in these four cell clusters from the supplementary materials in reference [13] for subsequent analysis.
3. The reconstruction of the OL developmental trajectory indicated that OPC2 cells were the youngest cell type, followed by OPC1, Oligos3 and Oligos1 [13]. The subsequent analysis was based on the developmental trajectory (OPC2 → OPC1 → Oligos3 → Oligos1). In this study, we defined this developmental trajectory as four developmental stages: Stage 1 (OPC2), Stage 2 (OPC1), Stage 3 (Oligos3) and Stage 4 (Oligos1). The workflow of this study is shown in Fig. 1.

**Table 1:** The number of four cell types in the HC and MDD group

Group	OPC2 stage1	OPC1 stage2	Oligos3 stage3	Oligos1 stage4
HC	312	793	1989	166
MDD	164	888	1632	70

### Identification of genes distinguishing the four developmental stages of OL

The GSE144136 dataset in “SRA” format was downloaded and converted into “fastq” format using FASTQ-dump in SRAToolkit. The “sampling” and “RRF” R packages were used as stratified sampling methods and for GRRF analysis, respectively. These analytic packages were used in R software (version 3.6.3, https://www.r-project.org). In the GRRF classifier, DEGs in four cell clusters based on Nagy et al. were validated by a fivefold cross validation method. The top-ranked genes of the four cell clusters were further subjected to partial least squares discriminant analysis (PLS-DA) to screen candidate genes, which obviously separated the four cell clusters. PLS-DA was analysed by the “MetaboAnalystR” R package [40]. PLS-DA enables the analysis of variable importance in projection (VIP) values, which emphasizes the importance of each variable during prediction.

### Identified genes distinguishing two adjacent developmental stages (stage 1 & stage 2, stage 2 & stage 3, stage 3 & stage 4)

Normally, developmental stages can be identified by stage-specific marker genes. To more precisely delineate developmental stages and enrich stage-specific marker genes, we detected genes that distinguish adjacent developmental stages via GRRF. Simultaneously, the discriminative ability of each classifier was measured by receiver operating characteristic (ROC) curves, and the area under the ROC curve (AUC) was calculated using the R package “ROCR”. We screened genes with top-ranked Gini scores, which were able to distinguish Stage 1 & Stage 2, Stage 2 & Stage 3, Stage 3 & Stage 4 and defined these genes as Group1, Group2, Group3.

### Identification genes distinguishing HC and MDD cases in four developmental stages

Each of the four developmental stages of the OL may play a role in the occurrence and development of depression, but to what extent each contributes is unknown. To assess the four developmental stages, we employed GRRF to establish predictive models based on DEGs between HC and MDD cases, and ROC curves were rendered on four cell types using the R package “ROCR”.

### Matching of candidate genes with published MDD-related databases and pathways

To improve the correlation between candidate genes and depression, we established a candidate gene list of depression-related genes through three approaches to facilitate the selection of candidate genes.

Approach 1: selecting genes associated with depression from the ingenuity pathways.

analysis (IPA) database (https://www.bilibili.com/video/av540856330/).

Approach 2: selecting genes associated with depression from the publicly available.

Database PsyGeNET http://www.psygenet.org/web/PsyGeNET/menu;jsessionid = 1t7yjgsorwmwz1ta1fv2k1yoib).

Approach 3: selecting genes from pathways closely related to depression.

(1) PATHWAY: hsa04726 Serotonergic synapse-Homo sapiens (human).

(https://www.kegg.jp/dbget-bin/www_bget?pathway+hsa04726).

(2) PATHWAY: hsa04722 Neurotrophin signaling pathway-Homo sapiens (human).

(https://www.kegg.jp/dbget-bin/www_bget?pathway+hsa04722).

(3) PATHWAY: hsa04080 Neuroactive ligand-receptor interaction-Homo sapiens (human).

(https://www.kegg.jp/dbget-bin/www_bget?pathway+hsa04080).

(4) PATHWAY: hsa04020 Calcium signaling pathway-Homo sapiens (human).

(https://www.kegg.jp/dbget-bin/www_bget?pathway+hsa04020).

(5) PATHWAY: hsa04915 Estrogen signaling pathway-Homo sapiens (human).

(https://www.kegg.jp/dbget-bin/www_bget?pathway+hsa04915).

(6) PATHWAY: hsa04010 MAPK signaling pathway-Homo sapiens (human).

(https://www.kegg.jp/dbget-bin/www_bget?pathway+hsa04010).

(7) PATHWAY: hsa04014 Ras signaling pathway-Homo sapiens (human).

(https://www.kegg.jp/dbget-bin/www_bget?pathway+hsa04014).

(8) PATHWAY: hsa04630 JAK-STAT signaling pathway-Homo sapiens (human).

(https://www.kegg.jp/dbget-bin/www_bget?pathway+hsa04630).

(9) PATHWAY: hsa04750 Inflammatory mediator regulation of TRP channels-Homo sapiens (human) (https://www.kegg.jp/dbget-bin/www_bget?pathway+hsa04750).

The selected depression-related genes from the IPA database, PsyGeNET and MDD-related pathways are listed in Additional file 1: Tables S1-S3, and the union of the two databases and pathways (Hereafter, the union of the two databases and pathways is designated as Union) is listed in Additional file 1: Table S4.

### Animals

The animal study was carried out using male C57BL/6 mice (n = 20) weighing 16–18 g purchased from the Company of Experimental Animals of Hunan Slack King (Hunan, China). Before the experiment, the mice were acclimated to the laboratory environment for one week. All mice were maintained under standard laboratory conditions with a 12-h light/dark cycle (lights on at 08:00), 22 ± 2 °C, and relative humidity 45%-55% and had free access to food and water. All animal care and experimental procedures were in accordance with the Association for Assessment and Accreditation of Laboratory Animal Care (AAALAC) Guidelines and the National Institutes of Health (NIH) Guide for the Care and Use of Laboratory Animals.

### Chronic unpredictable mild stress (CUMS) model

Mice were randomly divided into two groups: the CUMS group (n = 10) and the Control group (n = 10). The CUMS model was based on a previous study and was slightly modified [41]. The CUMS group was exposed to nine kinds of mild stressors for 4 weeks: food deprivation for 24 h, water deprivation for 24 h, damp sawdust for 24 h, 45° tilted cages for 24 h, swimming in ice water for 5 min, swimming in hot water at 45 °C for 5 min, tail clamping for 5 min, day and night reversal and pushing and squeezing. The CUMS group received one type of stress per day, all of which were applied randomly, and the same type of stress was not applied for two consecutive days.

### Body weight and behavioural tests

The body weights of mice in both the Control group and the CUMS group were measured before and after CUMS. Three behavioural tests were performed, including the sucrose preference test (SPT), forced swimming test (FST) and open field test (OFT). Anhedonia, an important clinical symptom of depression, was measured by SPT, and SPT was carried out as described in a previous study [42]. The FST is based on the assumption that when an animal is in a container filled with water and initially tries to escape but ends up staying still, the length of time it stays motionless reflects the degree of behavioural despair [43]; this test was implemented as described previously [44]. The OFT is a method to evaluate the autonomous and inquiry behaviour of rodents in new environments. The experimental apparatus consisted of an open field reaction box (50 cm*50 cm*50 cm) and an automatic video tracking system (Ethovision XT 11.5). Each mouse was placed in the centre of the box, and the frequency of rearing and distance travelled in the box were recorded during a 5-min session in a quiet environment.

### Sample collection

After all behavioural tests and data analyses, five mice in the Control group and CUMS group were anaesthetized with sodium pentobarbital (60 mg/kg, i.p.) and internally infused with 4% paraformaldehyde. Whole brain tissues were collected, postfixed with 4% paraformaldehyde for 24 h, embedded in paraffin and sectioned at 5 μm thickness for the following fluorescence in situ hybridization (FISH) assay.

### FISH assay and statistical analysis

The probes used in the FISH assay were synthesized by GenePharma (Shanghai, China), and the sequences and modifications are listed in Table 2. The FISH assay was performed according to the protocol provided by the RNA FISH Kit (GenePharma, #F2220). Images were collected using a fluorescence microscope (Olympus BX51, Japan), and the fluorescence intensity was analysed by ImageJ (National Institutes of Health, Bethesda, MD). GraphPad Prism (version 5.0, GraphPad, USA) was employed for statistical analysis and graphing. The data are presented as the mean ± standard error of the mean (SEM). A two-tailed unpaired t test was used for comparisons between two groups. Statistical significance was indicated by a p value < 0.05.

**Table 2:** The probes used in the FISH assay

Gene symbol	Sequence (5′-3′)	Modification
*Malat1*	(1) TTTAATCTACAAGGCCGACC	(5′-Cy3)
	(2) TCCACTAAGATGCTAGCTTG	(5′-Cy3)
	(3) ACATGCAATACTGCAGATC	(5′-Cy3)
*Pdgfra*	(1) AAATGGGACCTGACTTGGTG	(5′-FAM)
	(2) CCGGAGAGGAGAGTTAACAC	(5′-FAM)
	(3) GCCACGAGTCTAGAAAGACG	(5′-FAM)
*Mbp*	(1) AAAGAGGCGGATCAAGTGGG	(5′-FAM)
	(2) CGGGATTAAGAGAGGGTCTG	(5′-FAM)
	(3) ACCATGAGAAGTGGCCAGAG	(5′-FAM)

## Results

### Identification of genes distinguishing the four developmental stages of the OL

**Fig. 2:**
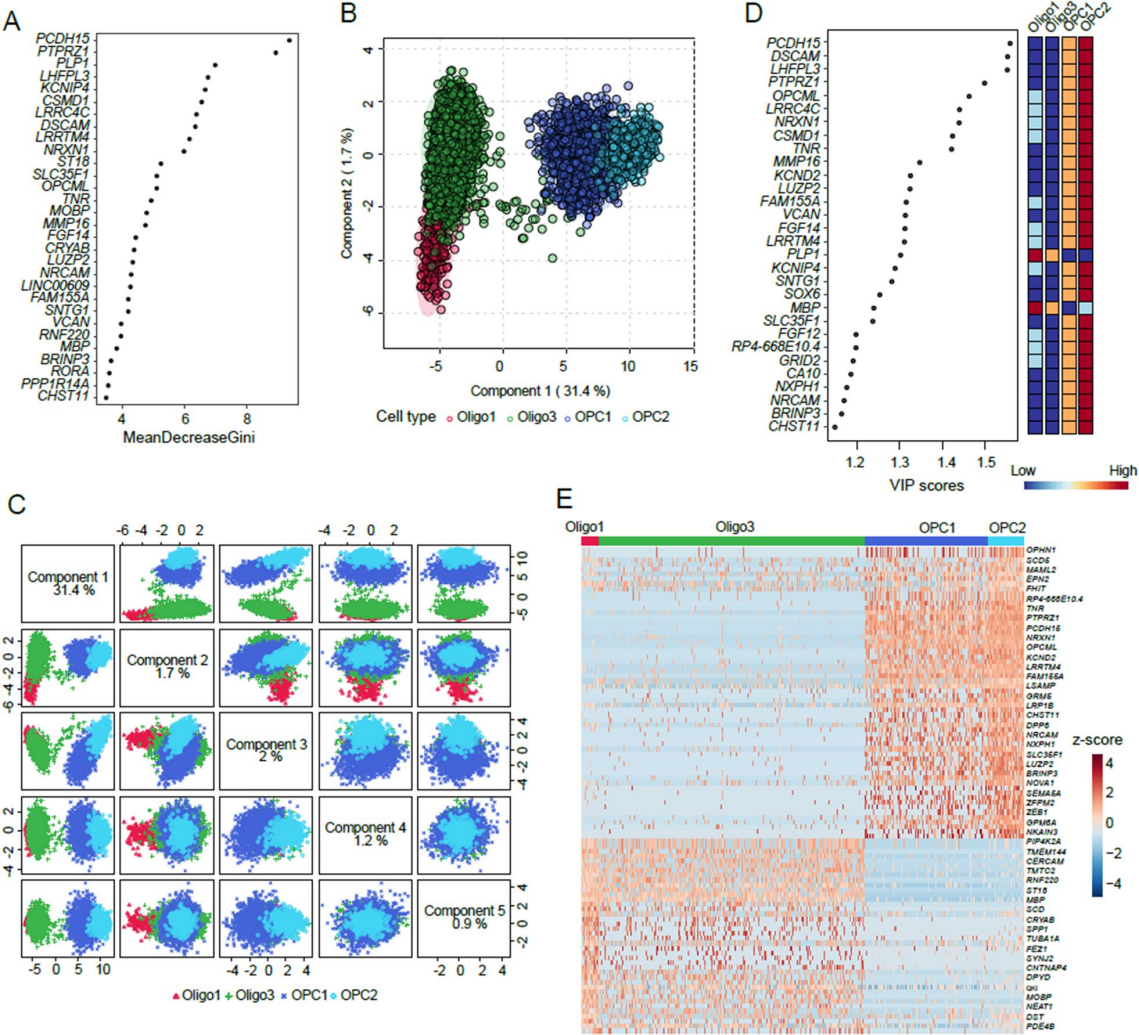
The top100 genes from GRRF model to distinguish four cell types. **A** Gene symbol and Gini scores of top 30 candidates. **B** PLS-DA score plots of four cell types based on top100 genes. **C** PLS-DA score plots of the top 5 components. **D** VIP scores of top 30 genes, with direction of gene expression shown on right panel. **E** Heatmap of top 100 genes among four cell types

To identify genes distinguishing the four developmental stages of the OL, we selected four cell types, OPC2 (Stage 1), OPC1 (Stage 2), Oligos3 (Stage 3), and Oligos1 (Stage 4), for subsequent analyses. By employing the GRRF algorithm and fivefold cross-validation method, classifiers based on DEGs were constructed. Gene symbols and Gini scores of the top 30 candidates are shown in Fig. 2A. We further screened 100 top-ranked genes based on Gini scores to detect candidate genes using PLS-DA. The PLS-DA score plots (Figs. 2B, C) indicated obvious separation between the four cell types, with the first component accounting for 31.4%. The VIP scores of the top 5 components of the top 48 candidates were greater than 1 (highlighted in bold in Additional file 1: Table S5), suggesting that the candidate genes possess high classification ability. Figure 2D shows the top 30 gene symbols and VIP scores with the direction of gene expression among the four cell types shown in the right panel. The heatmap (Fig. 2E) exhibits the changes in the top 100 genes among the four cell types. Genes with the top 100 Gini scores in the GRRF classifier are listed in Additional file 1: Table S6. Overall, these results demonstrate that the top 100 genes are capable of distinguishing between the four developmental stages. The screened genes might be important supplements for developmental stage-specific markers.

### Identification of genes distinguishing two adjacent developmental stages

**Fig. 3:**
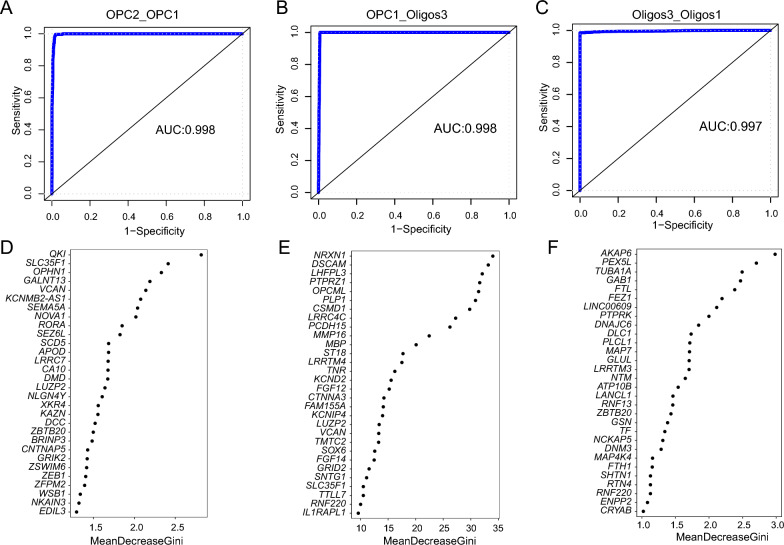
Constructing GRRF model for distinguishing two adjacent stages. ROC plot in **A** Stage1/ Stage2, **B** Stage2/ Stage3, **C** Stage2/ Stage3. Top 30 genes according to ranking Gini scores in **D** Stage1/ Stage2, **E** Stage2/ Stage3, **F** Stage2/ Stage3

When cells transition from one developmental stage to another, certain characteristics are observed, in addition to the regulation and expression of some key genes [10]. To identify these key genes, the GRRF method was employed to construct predictive models to distinguish every two adjacent developmental stages (Stage 1 & Stage 2, Stage 2 & Stage 3, Stage 3 & Stage 4). The ROC curves (Fig. 3A–C) showed the performance of the GRRF method and a high accuracy, with the AUC equalling 0.998, 0.998 and 0.997 when distinguishing Stage 1 & Stage 2, Stage 2 & Stage 3, Stage 3 & Stage 4, respectively. Figure 3D–E show the gene symbols and Gini scores of the top 30 genes corresponding to two adjacent developmental stages. Group 1, Group 2 and Group 3 of the top 100 genes were listed in Additional file 1: Tables S7–S9. The genes identified are likely to be key factors that propel cells to the next developmental stage, as well as potential complementary molecular markers that distinguish each stage of development.

**Fig. 4:**
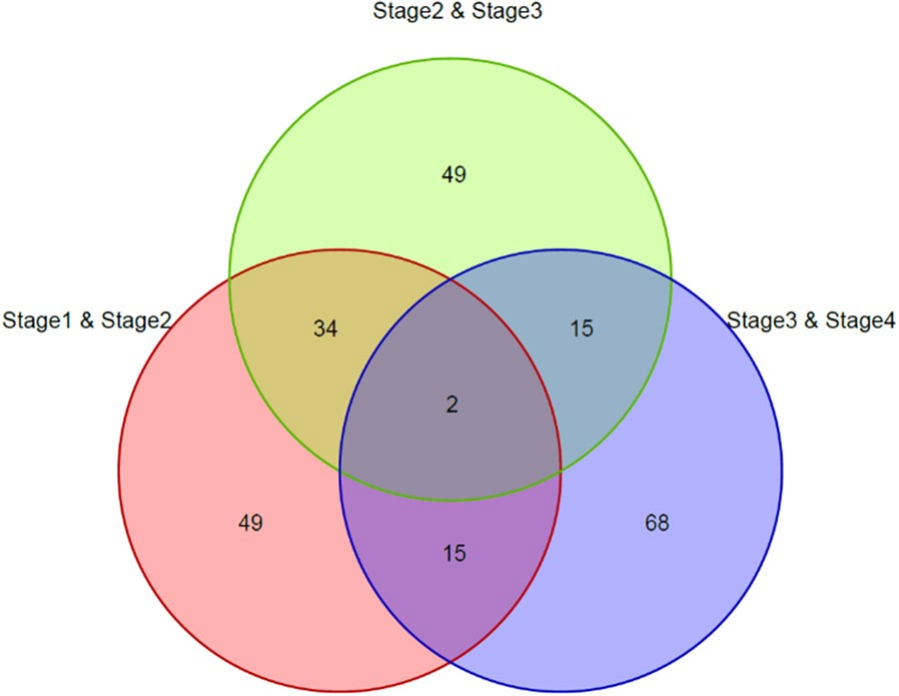
Venn diagram of top100 genes distinguishing two adjacent stages (Stage1&Stage2, Stage2&Stage3, Stage3&Stage4)

To explore the biological relevance of the top 100 genes in the three groups (Group 1, Group 2, and Group 3), we performed gene ontology (GO) and Kyoto Encyclopedia of Genes and Genomes (KEGG) analyses and obtained the following results. Group 1 was found to be involved in cell adhesion molecules, axon guidance and microRNAs in cancer, while Group 2 and Group 3 were found to be involved in cell adhesion molecules, glutamatergic synapses, neuroactive ligand-receptor interactions and mineral absorption, endocytosis, and GABAergic synapses, respectively. The KEGG pathways in which each group were found to be involved in are listed in Table 3, and GO analysis of the top 100 genes in the three groups is listed in Additional file 1: Tables S10–S12. We found that both Group 1 and Group 2 are involved in the cell adhesion molecule signalling pathway, while Group 2 and Group 3 are involved in different signalling pathways. Further analysis revealed that the intersection of Group 1 and Group 2 was 36, and the intersection of Group 2 and Group 3 was 17 (Fig. 4). This indicates that the conversion between Stage 1, Stage 2 and Stage 3 is relatively similar, while the conversion between Stage 3 and Stage 4 is quite different from the previous transitions.

**Table 3:** KEGG pathways three groups (Stage1&Stage2, Stage2&Stage3, Stage3&Stage4) of top 100 genes engaged in

Group	KEGG pathway term	Gene symbol
Stage1/Stage2	hsa04514:Cell adhesion molecules (CAMs)	*NTNG1, VCAN, ALCAM, NLGN4X, NEGR1, NRXN3, NRCAM, LRRC4C*
	hsa04360:Axon guidance	*NTNG1, SEMA5A, DCC, LRRC4C*
	hsa05206:MicroRNAs in cancer	*PDGFRA, ZEB1, MMP16, TNR, ZFPM2*
Stage2/Stage3	hsa04514:Cell adhesion molecules (CAMs)	*CLDN11, VCAN, NRXN1, CNTN1, NRCAM, LRRC4C*
	hsa04724:Glutamatergic synapse	*GRM5, GRM7, GRIK1, DLGAP1, GRIK2*
	hsa04080:Neuroactive ligand-receptor interaction	*GRM5, GRID2, GRM7, GRIK1, GRIK2*
Stage3/Stage4	ptr04978:Mineral absorption	*TF, FTH1, FTL*
	ptr04144:Endocytosis	*DNM3, ZFYVE16, DNAJC6, PSD3, MVB12B*
	ptr04727:GABAergic synapse	*PLCL1, GLUL, GPHN*

### Identification of candidate genes in the four developmental stages for distinguishing HC and MDD

**Fig. 5:**
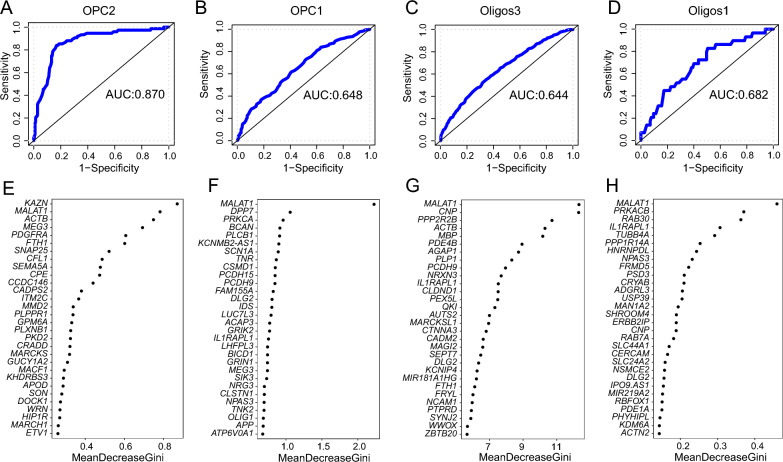
Constructing GRRF model from DEGs in four cell types distinguishing HC and MDD. ROC plot in **A** OPC2, **B** OPC1, **C** Oligos3, **D** Oligos1. Top 30 genes according to ranking Gini scores in **E** OPC2, **F** OPC1, **G** Oligos3, **H** Oligos1

Each of the four developmental stages of the OL may play a role in the occurrence of depression. To determine which of the four developmental stages is most closely associated with the onset of depression, we adopted the GRRF algorithm to construct disease predictive models based on the DEGs between HC and MDD cases. To screen genes distinguishing MDD cases from HC at each developmental stage, we selected the top 100 genes (Additional file 1: Tables S13-16) according to the Gini scores at each developmental stage. Figure 5A–D show that OPC2 exhibited the best predictive ability (AUC = 0.870), followed by Oligos1 (AUC = 0.682), OPC1 (AUC = 0.648) and Oligos3 (AUC = 0.644). Figure 5E–H show the gene symbols and importance scores of the top 30 genes in the four cell types. Surprisingly, the numbers of cells in the OPC2 (HC = 312, MDD = 164) and Oligos1 (HC = 166, MDD = 70) categories were much smaller than those in the OPC1 (HC = 793 MDD = 888) and Oligos3 (HC = 1989, MDD = 1632) categories; however, the former two possess significantly better predictive power than the latter two.

According to the original sequencing results, the number of cells in the OPC2 and Oligos1 categories was very small in the MDD group and HC group, accounting for less than 10%, without considering the technical systematic error. The number of cells in OPC1 and Oligos3 accounted for approximately 30% and 60%, respectively. We speculate that the conversion process from Stage 1 to Stage 2 was relatively fast, but the process from Stage 3 to Stage 4 was slow, resulting in more than half of the cells remaining in Stage 3. The results indicate that among the effects of the OL on depression, a few cells could play an important role in the occurrence of depression. The findings also highlight the importance of single-cell sequencing and provide a new perspective for studying diseases.

### Identification of *MALAT1* and *DLG2* by comparison with published MDD-related databases and pathways

**Fig. 6:**
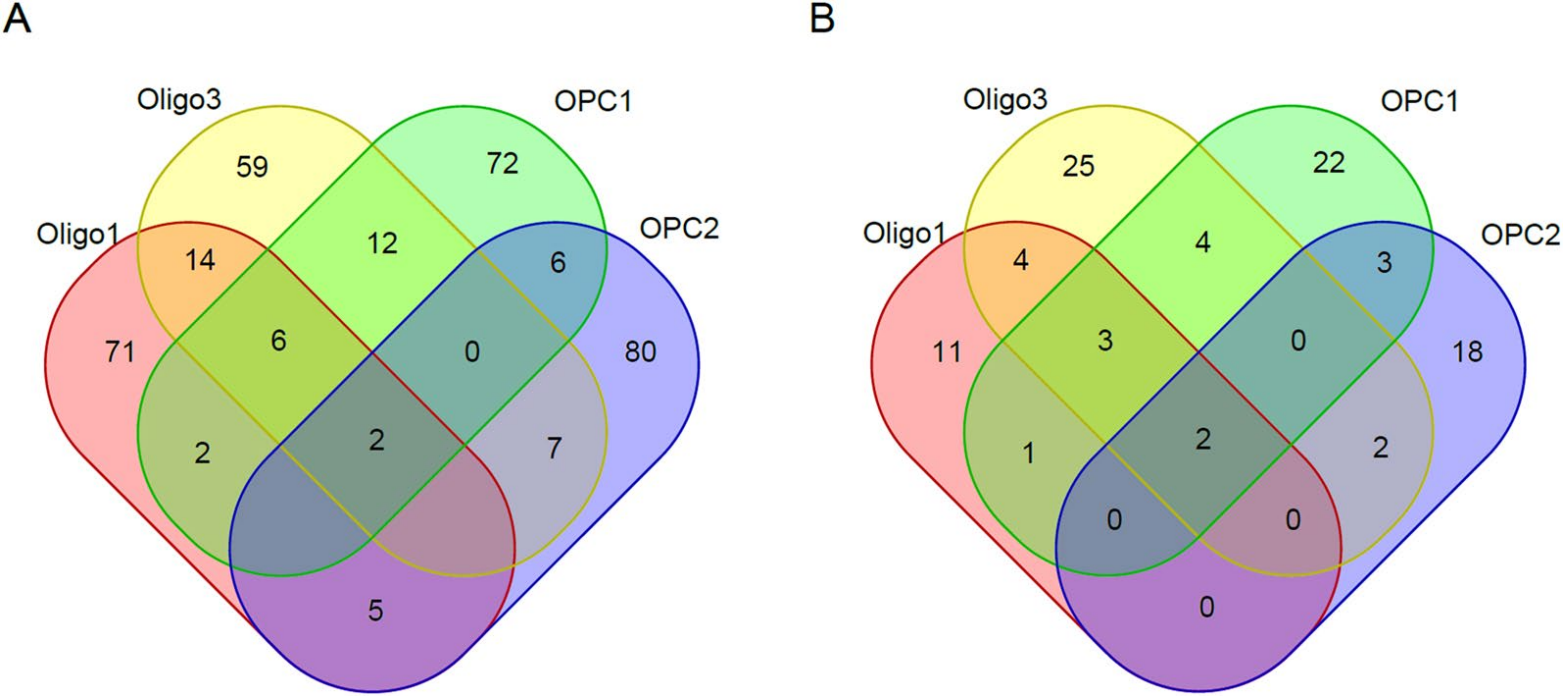
Venn diagram of top 100 genes distinguishing between HC and MDD from four cell types **A** Without retrieving in the list of candidate genes. **B** With retrieving in the list of candidate genes

To further compare our candidate genes with previously reported findings in MDD, we used the IPA database, the publicly available Database PsyGeNET and pathways closely related to depression. We retrieved the top 100 genes associated with the four developmental stages in the Union, and the number of overlapping genes of the four developmental stages was 25, 35, 40 and 21. The overlapping of the top 100 genes at each stage and in candidate gene list are listed in Table 4. Further analysis of the top 100 genes in the predictive model of four developmental stages revealed that two genes, namely, *MALAT1* and *DLG2*, appeared in all four stages (Fig. 6A). Interestingly, *MALAT1* and *DLG2* appeared simultaneously in the overlapping of the top 100 genes and Union at four stages (Fig. 6B).

**Table 4:**
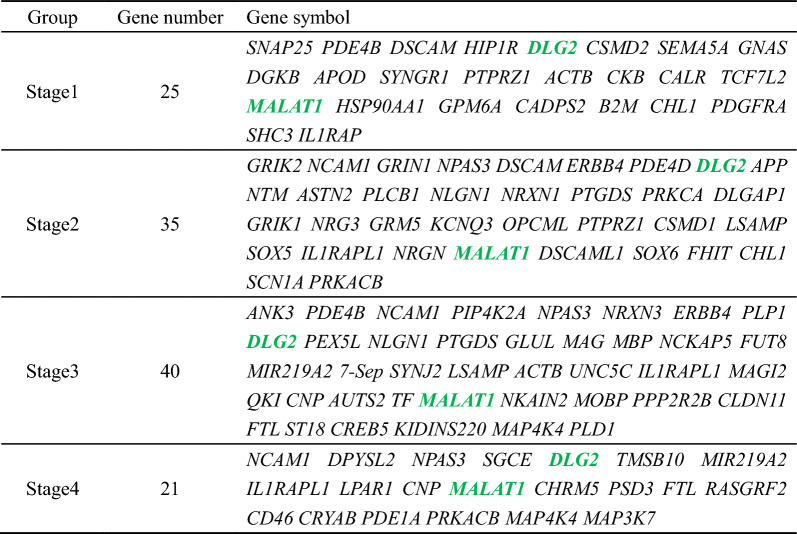
The intersection of MDD-related candidate gene list and top 100 genes distinguishing HC and MDD

*MALAT1* ranked second in Stage 1 and first in the other three stages. *MALAT1* has been widely studied in cancer, but little is known about its role in depression. *DLG2* is among the top 30 genes in Stage 2, Stage 3 and Stage 4. *DLG2* encodes the postsynaptic scaffolding protein *DLG2* (also referred to as PSD93), which interacts with NMDA receptors, potassium channels and cytoskeletal regulators. Genetic variation in the *DLG2* locus has been associated with a variety of psychiatric disorders. In view of the high ranking of *MALAT1*, we hypothesized that *MALAT1* may play an important role in the development of depression and selected it as a follow-up verification object.

### CUMS-susceptible mice showed lower body weight and obvious depressive-like behaviour

**Fig. 7:**
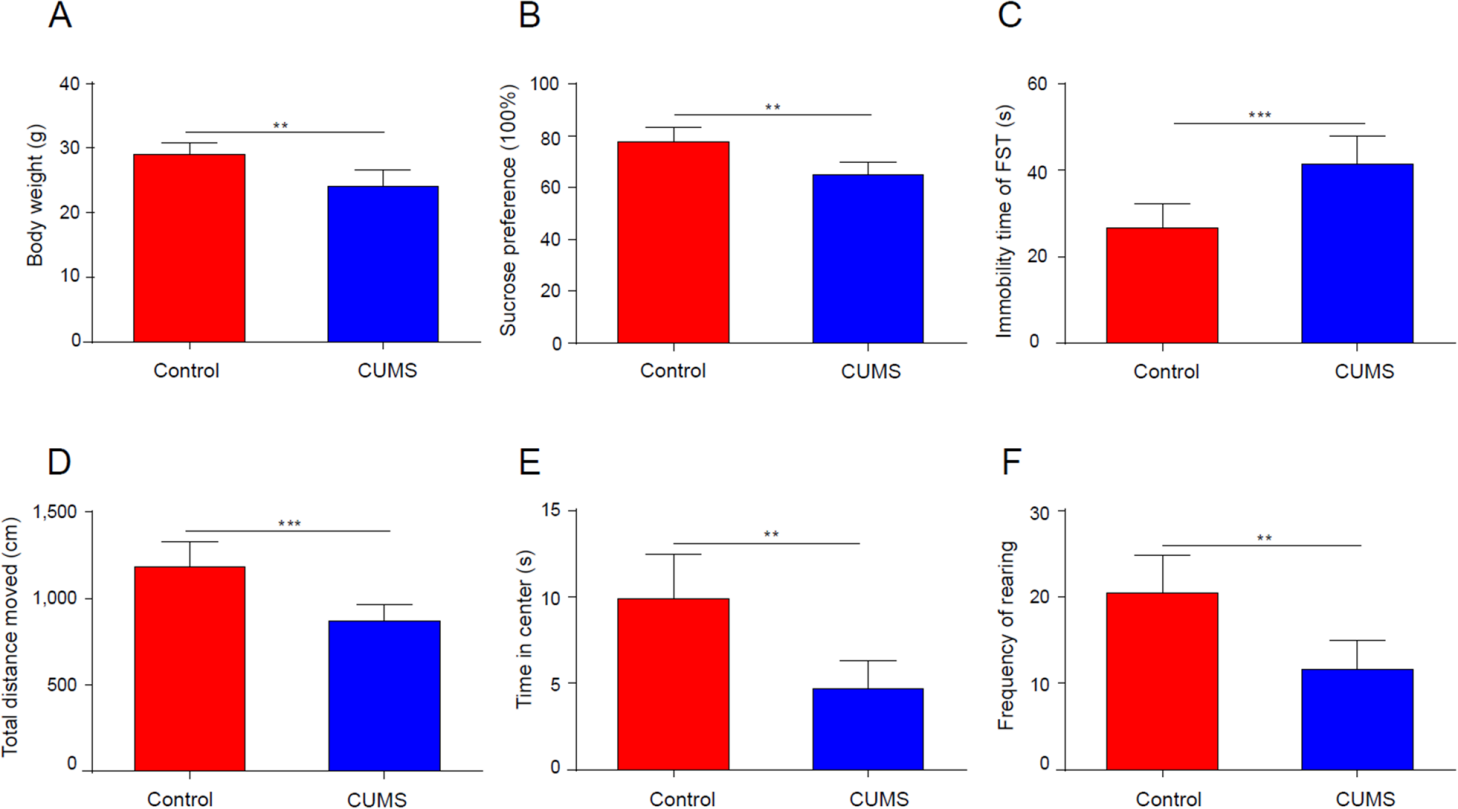
CUMS-susceptible mice showed lower body weight and obvious depression-like behavior. Effects of CUMS on body weight (**A**) and sucrose preference rate in the SPT (**B**); immobility time in the FST (**C**); total distance moved (**D**), time in the center (**E**), and rearing frequency (**F**) in the OFT. *P < 0.01, **P < 0.05, ***P < 0.001 vs. the Control group

Due to the difficulty of obtaining the human prefrontal cortex, we established a mouse CUMS model, which is recognized as reliable, practical, and widely employed to study the mechanism of depression. After 4 weeks of CUMS, mice in the CUMS group (n = 6) showed lower body weight than mice in the Control group [t(12) = 4.233, **P < 0.01, Fig. 7A] and obvious depression-like behaviour, including decreased sucrose preference rate [t(12) = 3.979, **P < 0.01, Fig. 7B]; total distance moved [t(12) = 4.639, ***P < 0.001, Fig. 7D], time in centre [t(12) = 4.305, **P < 0.01, Fig. 7E], frequency of rearing [t(12) = 4.14, **P < 0.01, Fig. 7F] in OFT; and increased immobility time in the FST [t(12) = − 4.571, ***P < 0.01, Fig. 7C]. The results indicated that mice with depression-like behaviour were successfully selected.

### Validation of *Malat1* expression in oligodendrocytes at different developmental stages in the PFC

**Fig. 8:**
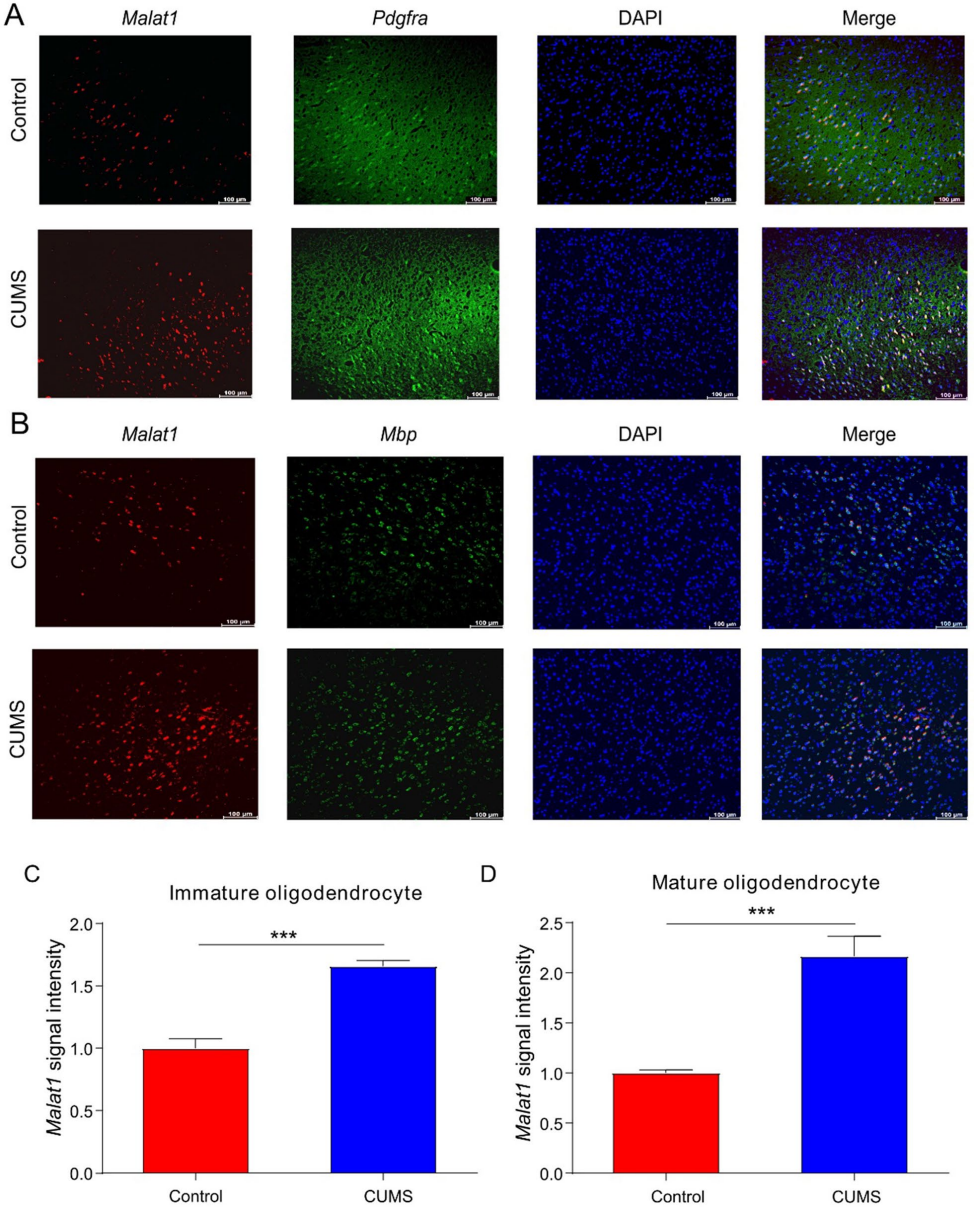
*Malat1* up-regulated in both immature and mature oligodendrocytes. Representative images of FISH assays of *Malat1* in immature (**A**) and mature (**B**) oligodendrocytes in the PFC, respectively. Increased *Malat1* signal intensity in immature (**C**) and mature (**D**) oligodendrocytes in the PFC, respectively. Nuclei were stained with DAPI; *Malat1* was labeled with Cy3, *Pdgfα* and *Mbp* were labeled with FAM. Scale bars represent 50 μm. The data were expressed as means ± SEM (n = 5). **P < 0.05 vs. the Control group

Our analysis shows that the expression of *MALAT1* was increased in oligodendrocytes at different developmental stages in the PFC. To validate this prediction, we conducted FISH assay with PFC slices from both the Control group and the CUMS group. *Pdgfα* and *Mbp* were selected as markers of immature oligodendrocytes and mature oligodendrocytes, respectively. FISH assay showed that *Malat1* expression was higher in both immature [t(8) = − 4.210, **P < 0.01, Fig. 8A and C] and mature [t(8) = − 4.877, **P < 0.01, Fig. 8B and D] oligodendrocytes in the CUMS group than in the Control group. The experimental results were consistent with the prediction, indicating that *Malat1* in oligodendrocytes at different developmental stages in the PFC is associated with the occurrence of depression; however, the specific mechanism needs to be further studied.

## Discussion

This study investigated the pathogenesis of depression from the perspective of oligodendrocyte development. The GRRF method was adopted to analyse the four developmental stages of the OL, and the following results were obtained. First, we screened the top-ranked genes distinguishing the four developmental stages based on DEGs, as well as those between two adjacent developmental stages. Then, we analysed the power of the four developmental stages to predict the occurrence of depression and found that the analysis of genes associated with the onset of OL development (Stage 1) possessed better predictive power than other developmental stages. Through comparison with the top-ranked candidate genes and subsequent FISH assay, we revealed that lncRNA *Malat1* might be closely related to the occurrence of depression.

ScRNA-seq technology has indicated that in brain tissues, gene expression patterns are cell-type specific in both dominant cell groups such as neurons and glial cells and in subtypes of neuronal cells or glial cells [45]. Generally, OL cells can be divided into OPCs and mature oligodendrocytes according to the degree of development. *PTGDS*, *PDGFRA*, *PCDH15*, *OLIG2*, and *OLG1* are common markers of OPCs, and *PLP1*, *MAG*, *MOG*, *MOBP*, and *MBP* are frequently-used markers of mature oligodendrocytes [10]. However, the division of these markers is not very rigorous; for instance, *PDGFRA* and *PCDH15* are only expressed in immature oligodendrocytes, while *OLIG2* and *SOX10* are expressed in both immature and mature oligodendrocytes [13]. The top-ranked genes identified included *PCDH15*, *PLP1*, *MAG*, *MBP* and other routine markers, and among the top 100 candidates, *PCDH15* ranked first, indicating the high feasibility of our prediction method.

By reviewing the single-cell gene expression profile in GSE144136, we found that three genes, including *PCDH15*, *DSCAM* and *PTPRZ1*, are only expressed in OPCs but not in neurons and are all associated with cell adhesion. *DSCAM* is located on human chromosome 21 and belongs to the immunoglobulin superfamily of cell adhesion molecules. It is unquestionable that *DSCAM* plays a role in regulating cell recognition, neural circuit formation, and the delamination of neurons in the developing midbrain [46, 47]. Furthermore, Amano found an association between increased *DSCAM* expression and bipolar disorder in a genetic screen of patients with bipolar disorder. *PTPRZ*, a protein tyrosine phosphatase, is mainly expressed in astrocytes, oligodendrocyte precursor cells (OPCs), and immature and mature oligodendrocytes of the developing and adult nervous system [48]. This receptor binds to the cell adhesion molecules on the surface of OPCs and participates in the proliferation and differentiation of OPCs [49]. Studies have shown that *PTPRZ* affects the balance between OPC proliferation and maturation by forming a complex with *CNTN1* on the surface of OPCs, inhibiting their proliferation and promoting their transformation into mature oligodendrocytes [50]. Our analysis showed that the screened genes could be used as potential developmental markers of OL.

After the 1950s, with the development of intracellular recording methods and histochemistry, our understanding of glial cells became more comprehensive. At present, OL cells are generally classified as mature or immature. Our analysis divided the development of the OL into four stages and identified the key genes that distinguish each of the two adjacent stages. Quaking (*qki*), encoding a conserved RNA-binding protein QKI, ranked first in Group 1. Reportedly, *QKI* plays a specific role in myelin defects in the aetiology of psychiatric disorders and is critical to the myelination decision of the OL in MDD suicide victims [51]. Studies have shown that *Qki* participates in the regulation of myelin lipid homeostasis, and deletion of *Qki* in oligodendrocytes did not affect oligodendrocyte survival but resulted in rapid demyelination in adult mice within one week and progressive neurological dysfunction [52]. Group 2, distinguishing OPC1 (Stage 2) and Oligos3 (Stage 3), promotes the transformation of immature oligodendrocytes into mature oligodendrocytes. Our results show that *PCDH15*, *PLP1* and *MBP* were all within the top 10 of Group 2, consistent with previous studies wherein *PCDH15* was found to be highly expressed in late OPCs and was a marker of immature oligodendrocytes [53], and *PLP1* and *MBP* are markers of mature oligodendrocytes [54].

Oligodendrocyte development is also accompanied by changes in the expression of neurotransmitter receptors on the cell surface. KEGG pathway analysis showed that Group 2 was involved in the glutamatergic synapse pathway, while Group 3 participated in the GABAergic pathway. Bergles first reported that OPCs accepted the introduction of excitatory glutamatergic through AMPA receptors [55]. Subsequently, Lin found that OPCs expressed GABA receptors in response to GABAergic input [56]. Ablation of OPCs in the prefrontal cortex of adult mice altered AMPA receptor membrane trafficking, impaired excitatory glutamatergic neurotransmission and extracellular glutamate uptake, and ultimately led to depressive-like behaviour in mice [57]. OPCs can form synaptic complexes with hippocampal interneurons. Photostimulation of OPCs stimulates GABA release and affects hippocampal excitatory-inhibitory balance, resulting in anxiety-like behaviours in mice [58].

For a long time, OL cells were regarded as a source of myelinating cells. Recent research has shown that OL cells have other roles, such as regulating the function of neurons and astrocytes, ultimately affecting behaviour, responding to central nervous system damage and acting as innate immune cells [59]. Recent studies have shown that the loss of OPCs in the prefrontal cortex alters glutamate energy signalling and promotes depression-like behaviour in mice [57]. Nagy et al. also found that OPCs played a significant role in the occurrence of depression in humans [13]. Recently, an interesting discovery showed that the immune system utilizes OPCs to maintain its immune response in the demyelination state [60, 61]. Both OPCs and oligodendrocytes act as antigen-presenting cells and activate CD8^+^ T cells in humans and mice [62, 63]. Neuroinflammation is a well-known molecular mechanism of depression, and we hypothesize that OPCs may be involved in neuroinflammation leading to depression.

The OPC2 subcellular type, the initiation stage of the OL, had the best predictive power, and we infer that there may be two reasons. On the one hand, OPCs account for approximately 5% of the total number of mature brains and retain the ability to self-proliferate throughout life [9]. On the other hand, although the primary function of OPCs is to proliferate and differentiate into mature oligodendrocytes, OPCs can also differentiate into astrocytes [64] and neurons [65] at certain developmental stages and brain regions. Depression is associated with neurogenesis and neuroplasticity, and the continued proliferation and transformation of OPCs into neurons or glia may play a compensatory role.

The LncRNA *MALAT1*, also known as *NEAT2*, was first discovered by Ji [66]. *MALAT1* is dominantly found in nuclear speckles and was highly conserved during mammalian evolution. It is widely expressed in normal mammalian tissues and has elevated levels in most malignant tissues [67]. Recently, an increasing number of studies have also linked *MALAT1* to neurological disorders such as schizophrenia (SZ), Alzheimer’s disease (AD) and neuropathic pain. The level of *MALAT1* in peripheral blood decreased in patients with SZ [68]. *MALAT1* positively regulates the expression of *CDK5R1* and affects the occurrence of AD [69], and regulation of the miR-129-5p/HMGB1 axis causes the occurrence of neuropathic pain in a chronic constriction injury model in rats [70]. Currently, there are few reports on the relationship between *MALAT1* and depression [71–73]. Our results provide a new target for the study of long noncoding RNAs in depression.

Unfortunately, there are four main limitations to this study. First, since the sample collection lasted 15 years, single nuclei could only be isolated from tissues, not single cells, and transcripts within the cytoplasm were lost; thus, the analysis could not be performed at the overall transcript level. Second, only the relationship between the development of the OL and the occurrence of depression was analysed, and the role of other cell types was ignored. In addition, brain samples from patients with depression are limited and extremely difficult to collect, and therefore, we verified the candidate gene *Malat1* in animal models. It is more logical to perform this analysis in human samples. Finally, this manuscript is a reanalysis of data obtained in a previous work of Nagy Corina’s group [13], and is not a replication or confirmation. We conducted a correlation analysis of single-nucleus transcriptomics data of oligodendrocyte lineage in female samples (HC = 18, MDD = 20), also generated by Nagy Corina’s group [74]. The results showed that, 1) the pseudotime trajectory of oligodendrocyte lineage in female samples was similar to that in male samples, and 2) *Malat1* also distinguished HC and MDD at different developmental stages of oligodendrocyte in female samples (Additional file 1: Table S17, S18 and Figure S1-S4).

## Conclusion

This study investigated the mechanism of MDD from the perspective of OL development at the single-cell level. We adopted a GRRF algorithm to screen critical candidate genes in single-cell sequencing data processing. The results demonstrate that the initiation developmental stage of the OL was a better predictor of the occurrence of depression than other developmental stages, and that the lncRNA *Malat1* might be closely related to the occurrence of depression. Our work highlights the importance of single-cell sequencing in mental disorders and provides a novel direction for research on the occurrence of MDD.

### Supplementary Information

**Additional file 1: ** Figure S1：Oligodendrocyte lineage cell clusters of 38 female HC and MDD visualized by UMAP. (A): Colors indicate a female subject (HC or MDD). (B): Colors indicate cell types. Each dot represents one nucleus. HC: healthy control; MDD: major depressive disorder; UMAP: uniform manifold approximation and projection. Figure S2：Pseudotime trajectory (Monocle analysis) of the Oligodendrocyte lineage.(A): Cells are colored based according to the cell type; (B): Cells are colored based according to the predicted pseudotime. Figure S3：Constructing GRRF model for distinguishing two adjacent developmental stages. ROC plot in (A) OPC1/OPC2, (B) OPC2/Oligos1, (C) Oligos1/Oligos2, (D) Oligos2/Oligos3. Top 30 genes and Gini scores in (E) OPC1/OPC2, (F) OPC2/Oligos1,(E) Oligos1/Oligos2, (F) Oligos2/Oligos3. Figure S4：Constructing GRRF model from DEGs in four cell types distinguishing HC and MDD. ROC plot in (A) OPC1, (B) OPC2, (C) Oligos1, (D) Oligos2, (E) Oligos3. Top 30 genes and Gini scores in (F) OPC1, (G) OPC2, (H) Oligos1, (I) Oligos2, (J) Oligos3. Table S1: Depression-associated genes in IPA. Table S2: Depression-associated genes in PsyGeNET. Table S3: Depression-associated genes in MDD-related pathways. Table S4: The union of supplementary1&2&3 (Union). Table S5: VIP scores of the top 5 components of the top 100 genes distinguishing four development stages. Table S6: Genes with top 100 Gini scores in four stages. Table S7: Top 100 genes distingush between Stage1&Stage2. Table S8: Top 100 genes distingush between Stage2&Stage3. Table S9: Top 100 genes distingush between Stage3&Stage4. Table S10: GO analysis of top 100 genes in Stage1&Stage2 (Group1). Table S11: GO analysis of top 100 genes in Stage2&Stage3 (Group2). Table S12: GO analysis of top 100 genes in Stage3&Stage4 (Group3). Table S13: Gini scores of top 100 genes of HC and MDD in Stage1. Table S14: Gini scores of top 100 genes of HC and MDD in Stage2. Table S15: Gini scores of top 100 genes of HC and MDD in Stage3. Table S16: Gini scores of top 100 genes of HC and MDD in Stage4. Table S17: Number of DEGs for 5 cell types. Table S18: Number of DEGs for developmental stage of different cell types.

## Data Availability

All the necessary data are included within the article. Further data will be shared by request.

## Abbreviations

AD: Alzheimer’s disease
AUC: Area under the curve
CUMS: Chronic unpredictable mild stress
CNS: Central nervous system
GRN: Gene regulatory network
GRRF: Guided regularized random forest
RF: Random forest
FISH: Fluorescence in situ hybridization
FST: Forced swimming test
HC: Healthy controls
IPA: Ingenuity pathways analysis
MDD: Major depressive disorder
OFT: Open field test
OL: Oligodendrocyte lineage
OPCs: Oligodendrocyte progenitor cells
PLS-DA: Partial least squares discriminant analysis
RNA-seq: RNA-sequencing
ROC: Receiver operating characteristic
SPT: Sucrose preference test
SZ: Schizophrenia
scRNA-seq: Single-cell RNA sequencing
