# Supplementary material for: Single-nucleus transcriptomic analysis reveals the relationship between gene expression in oligodendrocyte lineage and major depressive disorder
Source: J Transl Med. 2024 Jan 27;22:109. doi: 10.1186/s12967-023-04727-x (PMC10822185; doi:10.1186/s12967-023-04727-x)
Supplement: Supplementary file 1 — Additional file 1: Figure S1：Oligodendrocyte lineage cell clusters of 38 female HC and MDD visualized by UMAP. (A): Colors indicate a female subject (HC or MDD). (B): Colors indicate cell types. Each dot represents one nucleus. HC: healthy control; MDD: major depressive disorder; UMAP: uniform manifold approximation and projection. Figure S2：Pseudotime trajectory (Monocle analysis) of the Oligodendrocyte lineage.(A): Cells are colored based according to the cell type; (B): Cells are colored based according to the predicted pseudotime. Figure S3：Constructing GRRF model for distinguishing two adjacent developmental stages. ROC plot in (A) OPC1/OPC2, (B) OPC2/Oligos1, (C) Oligos1/Oligos2, (D) Oligos2/Oligos3. Top 30 genes and Gini scores in (E) OPC1/OPC2, (F) OPC2/Oligos1,(E) Oligos1/Oligos2, (F) Oligos2/Oligos3. Figure S4：Constructing GRRF model from DEGs in four cell types distinguishing HC and MDD. ROC plot in (A) OPC1, (B) OPC2, (C) Oligos1, (D) Oligos2, (E) Oligos3. Top 30 genes and Gini scores in (F) OPC1, (G) OPC2, (H) Oligos1, (I) Oligos2, (J) Oligos3. Table S1: Depression-associated genes in IPA. Table S2: Depression-associated genes in PsyGeNET. Table S3: Depression-associated genes in MDD-related pathways. Table S4: The union of supplementary1&2&3 (Union). Table S5: VIP scores of the top 5 components of the top 100 genes distinguishing four development stages. Table S6: Genes with top 100 Gini scores in four stages. Table S7: Top 100 genes distingush between Stage1&Stage2. Table S8: Top 100 genes distingush between Stage2&Stage3. Table S9: Top 100 genes distingush between Stage3&Stage4. Table S10: GO analysis of top 100 genes in Stage1&Stage2 (Group1). Table S11: GO analysis of top 100 genes in Stage2&Stage3 (Group2). Table S12: GO analysis of top 100 genes in Stage3&Stage4 (Group3). Table S13: Gini scores of top 100 genes of HC and MDD in Stage1. Table S14: Gini scores of top 100 genes of HC and MDD in Stage2. Table S15: Gini scores of top 100 genes of HC a [file 12967_2023_4727_MOESM1_ESM.zip › 12967_2023_4727_MOESM1_ESM/supplementary tables17-18 and supplementary figures1-4-V2.docx]

**Table S17:** Number of DEGs for 5 cell types.

| Cell type | OPC1 | OPC2 | Oligos1 | Oligos2 | Oligos3 |
| --- | --- | --- | --- | --- | --- |
| Number of DEGs | 144 | 3981 | 199 | 1640 | 171 |

**Table S18:** Number of DEGs for developmental stage of different cell types.

| Developmental stage | OPC1/  OPC2 | OPC2/  Oligos1 | Oligos1/  Oligos2 | Oligos2/  Oligos3 |
| --- | --- | --- | --- | --- |
| Number  of DEGs | 2964 | 3090 | 1466 | 1799 |


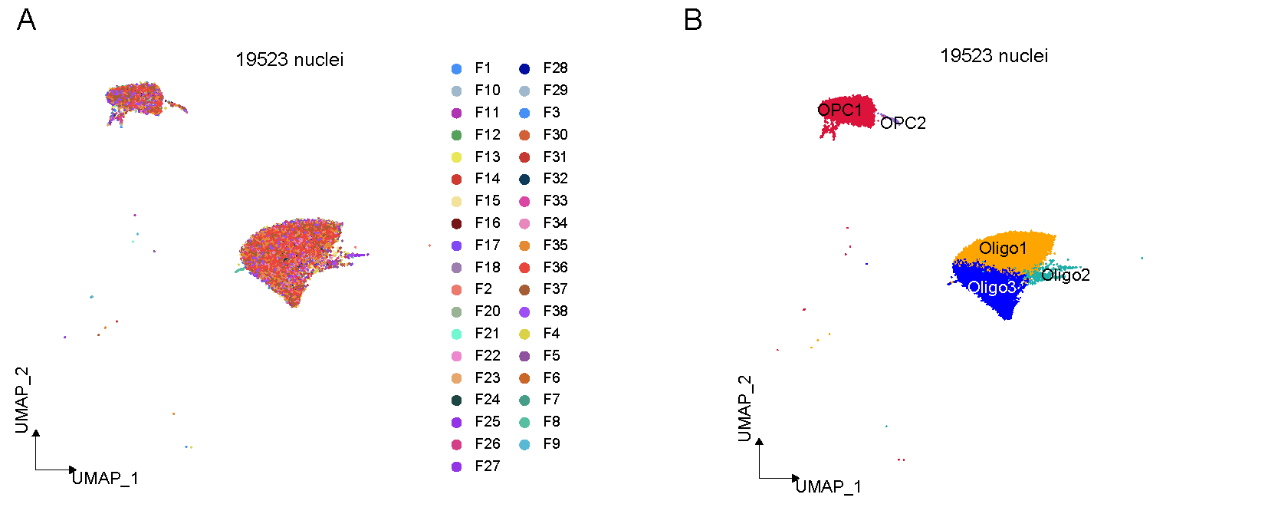


**Figure S1**：UMAP showing oligodendrocyte lineage cell clusters from 37 female HC and MDD. (**A**): Intergration of 37 female samples (HC or MDD). (**B**): Distribution of cell types on the UMAP. Each dot represents one nucleus. HC: healthy control; MDD: major depressive disorder; UMAP: uniform manifold approximation and projection.


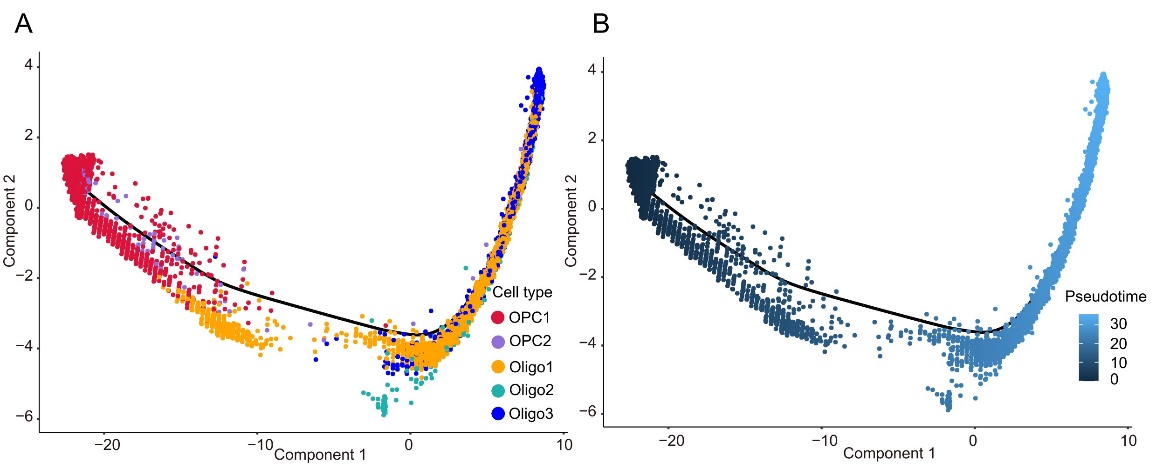


**Figure S2**：Pseudotime trajectory (Monocle2 analysis) of the oligodendrocyte lineage. (**A**): Cells are colored based according to the cell type. (**B**): Cells are colored based according to the predicted pseudotime.


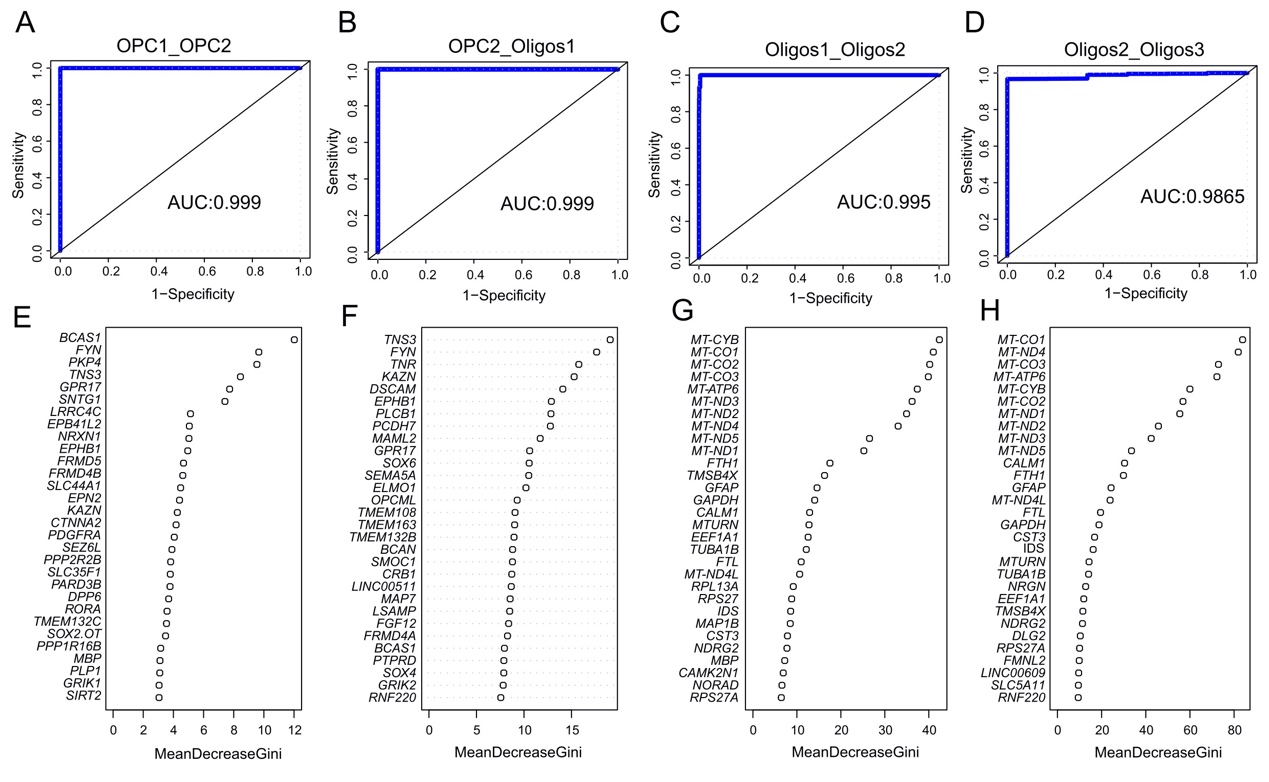


**Figure S3**：Constructing GRRF model for distinguishing two adjacent developmental stages. ROC plot in (**A**) OPC1/OPC2, (**B**) OPC2/Oligos1, (**C**) Oligos1/Oligos2, (**D**) Oligos2/Oligos3. Top 30 genes according to ranking Gini scores in (**E**) OPC1/OPC2, (**F**) OPC2/Oligos1,(**E**) Oligos1/Oligos2, (**F**) Oligos2/Oligos3.


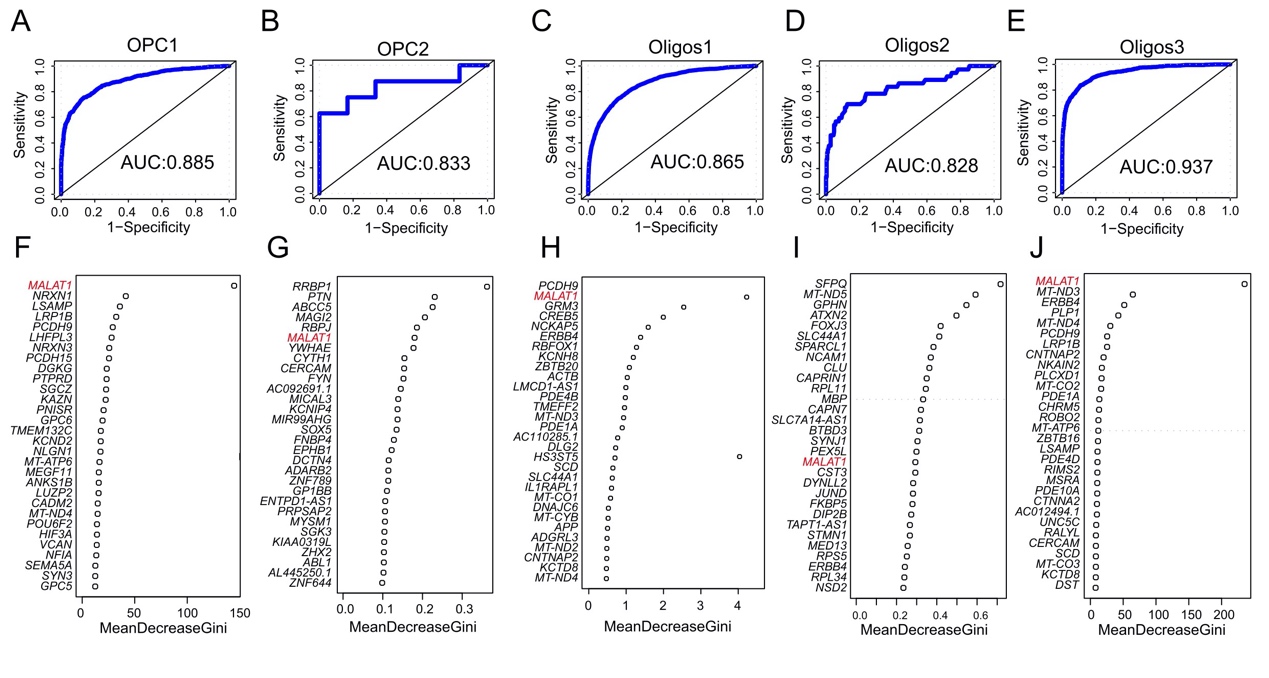


**Figure S4**：Constructing GRRF model from DEGs in four cell types distinguishing HC and MDD. ROC plot in (**A**) OPC1, (**B**) OPC2, (**C**) Oligos1, (**D**) Oligos2, (**E**) Oligos3. Top 30 genes according to ranking Gini scores in (**F**) OPC1, (**G**) OPC2, (**H**) Oligos1, (**I**) Oligos2, (**J**) Oligos3.
